# A Facile Fluorescent Visualization Method Based on Copper Clusters for Formaldehyde Detection

**DOI:** 10.3390/molecules30194022

**Published:** 2025-10-08

**Authors:** Jie Zou, Qing Chen, Guimin Mu, Miao Ma, Fang Yang, Mengtian Li, Fujian Xu, Hui Xia

**Affiliations:** Key Laboratory of Pollution Control Chemistry and Environmental Functional Materials for Qinghai-Tibet Plateau of the National Ethnic Affairs Commission, School of Chemistry and Environment, Southwest Minzu University, Chengdu 610041, Chinaxufujian@swun.edu.cn (F.X.)

**Keywords:** copper clusters, visual detection, formaldehyde, hydrogel stabilization

## Abstract

Establishing a simple and effective method for the visual detection of formaldehyde plays an important role in environmental emergency monitoring. In this work, L-cysteine-stabilized copper clusters were synthesized via a green, mild, and facile one-step preparation method. Through the optimization of reaction conditions, including reactant concentration and pH, the clusters exhibited stable red fluorescence. Upon exposure to formaldehyde, the fluorescence intensity of copper clusters gradually quenched with increasing formaldehyde concentration, enabling the development of a visual detection method that was successfully applied to analyze formaldehyde samples in air. Furthermore, by immobilizing the copper clusters into hydrogels, the visual detection performance and portability of the material were significantly enhanced. This method offers the advantages of simple preparation and rapid and accurate determination, demonstrating potential for semi-quantitative field detection of formaldehyde in emergency scenarios.

## 1. Introduction

Formaldehyde (HCHO) is a common and important industrial chemical used in the production of various compounds [1] which is regarded as one of the four major Volatile Organic Compound (VOC) pollutants in indoor air, and it was included in the list of carcinogens by the WHO [2,3]. So far, spectrophotometry [4], gas chromatography [5], high-performance liquid chromatography (HPLC) [6], Raman spectroscopy [7], and so on [8,9,10] have been introduced as methods for formaldehyde determination. However, these methods often require relatively complex pre-treatment processes and equipment, and the introduction of reagents during the procedures can lead to secondary pollution. Electrochemical detection methods based on semiconductor materials are simple and rapid, but they have certain limitations in terms of stability and selectivity [11,12]. In contrast, the colorimetric array visual detection method established using formaldehyde-sensitive dyes and semiconductor materials can enhance selectivity, yet its sensitivity still needs improvement [13,14]. Compared with those approaches, fluorescence (FL) methods offer some benefits, including being suitable for miniaturization and having high sensitivity and excellent stability and cost-effectiveness [15,16,17].

Since nanomaterials have excellent electrical, optical, thermal, and catalytic properties, they present the possibility of building sensors based on nanomaterials to monitor pollutants in the environment. For instance, Zhao et al. [18] synthesized a MnO_2_ nanosheet with oxidase-mimicking activity and constructed a universal fluorescent nano-platform to detect formaldehyde. Our previous work also proposed a simple colorimetric sensor array based on luminescent CdTe QDs, which can be used to visually detect formaldehyde in the air. Noble metal nanoparticles, such as Au NPs and Ag NPs, exhibit a unique surface plasmon resonance (SPR) phenomenon [19,20] and exhibit unique optical properties, as do metal nanoclusters (MNCs), due to their unique fluorescent properties and ultra-small size. For example, silver nanoclusters and copper nanoclusters with different protecting ligands have been employed for the construction of fluorescent visualization detection systems for formaldehyde [21,22,23]. Metal–organic framework materials (MOFs) [24,25] can sensitively detect formaldehyde due to their porosity, overhanging functional groups, and open metal sites. Although the materials mentioned above have shown excellent applications in formaldehyde detection, their high cost and toxicity still need to be improved, and the preparation of these materials remains relatively complex. Therefore, it is highly necessary to achieve the simple and rapid determination of formaldehyde using a sensing material that is easy to prepare and inexpensive.

**Scheme 1:**
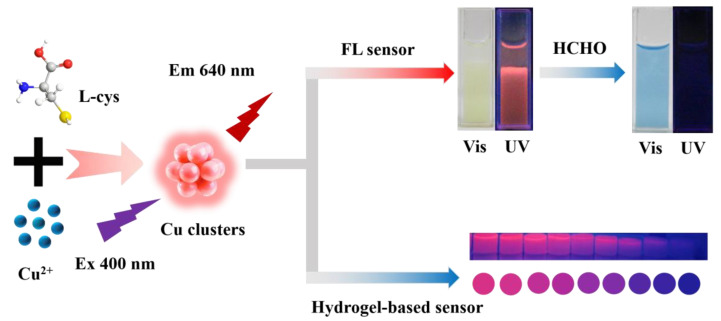
Schematic illustration of the synthetic steps of luminescent L-cys-Cu clusters for the FL and hydrogel-based visual detection of formaldehyde.

In our previous work, we achieved the fluorescence visualization sensing and detection of ammonia and sodium nitrite using copper clusters modified with different ligands and their composites [26,27]. This demonstrates that the advantages of copper clusters, such as easy availability, facile modification, and tunable fluorescence, make them well-suited for establishing simple identification methods for VOCs. Thus, this method establishes a novel formaldehyde detection approach utilizing fluorometric–colorimetric dual-mode sensing based on L-cysteine (L-cys)-modified copper clusters. Moreover, by integrating the material with hydrogels, it enables the portable and visually observable determination of samples, as illustrated in Scheme 1.

## 2. Results and Discussion

### 2.1. Characterization of Copper Clusters

**Figure 1:**
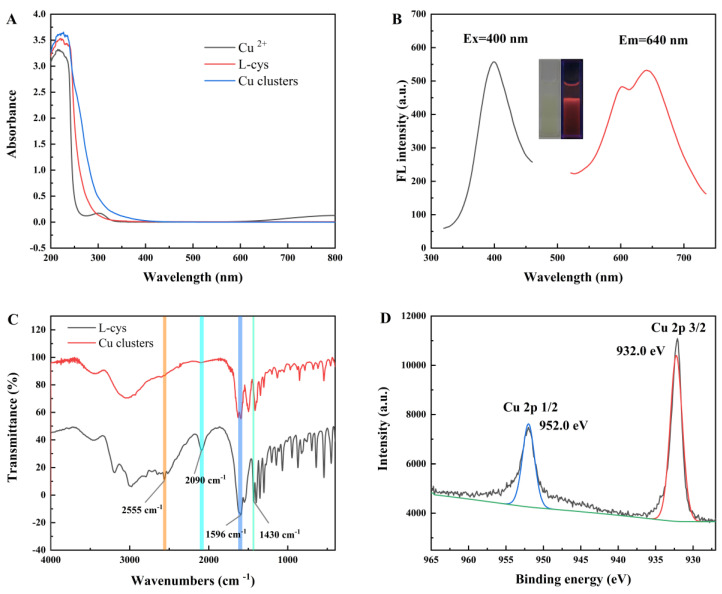
(**A**) UV-Vis spectra of Cu (NO_3_)_2_, L-cysteine, and copper clusters. (**B**) Excitation and emission spectra of copper clusters (Insert: photos of the copper clusters under visualization and UV light). (**C**) Comparative FT-IR spectra of L-cysteine and the synthesized copper clusters and (**D**) high-resolution XPS spectrum of Cu 2p copper clusters.

The characteristics of the aggregated luminescence of the synthesized copper clusters were investigated by fluorescence spectroscopy and UV-Vis spectroscopy. The absorption spectra of Cu (NO_3_)_2_ (a), L-cys (b), and copper clusters (c) are displayed in Figure 1A, respectively. Cu (NO_3_)_2_ exhibited a weak absorption peak around 300 nm, and L-cysteine showed no obvious peaks. The copper clusters had a weak absorption peak around 260 nm, exhibiting molecular-like optical properties, which is characteristic of Cu NCs [28]. Figure 1B shows the fluorescence spectrum of the Cu clusters; it has two strong emission peaks at 600 nm and 640 nm, with a large Stokes shift under the optimal excitation at 400 nm. To guarantee the sensitivity of the detection, 640 nm was chosen as the optimal emission wavelength. The structural characteristics of copper clusters were characterized using FT-IR spectroscopy. As shown in Figure 1C, which presents the FT-IR spectra of the ligand L-cys and the synthesized copper clusters, a comparative analysis reveals that the characteristic peak of the thiol group (-SH) at 2555 cm^−1^ disappears in the synthesized copper clusters. Given that the thiol group on L-cys possesses reducing properties, it can reduce Cu(II) to Cu(I) or Cu(0) and subsequently bind to it, indicating that the copper clusters are formed through Cu-S bond conjugation. In addition, the valence state of copper in the copper clusters was studied by XPS. As shown in Figure 1D, two obvious peaks appeared at 932.0 and 952.0 eV, which could correspond to the binding energies of the 2p 3/2 and 2p 1/2 electrons of Cu(0) or Cu(I). The absence of additional vibrational satellite peaks from 938 to 946 eV indicates the absence of Cu(II) in the copper clusters.

### 2.2. Optimization of Synthesis Conditions

**Figure 2:**
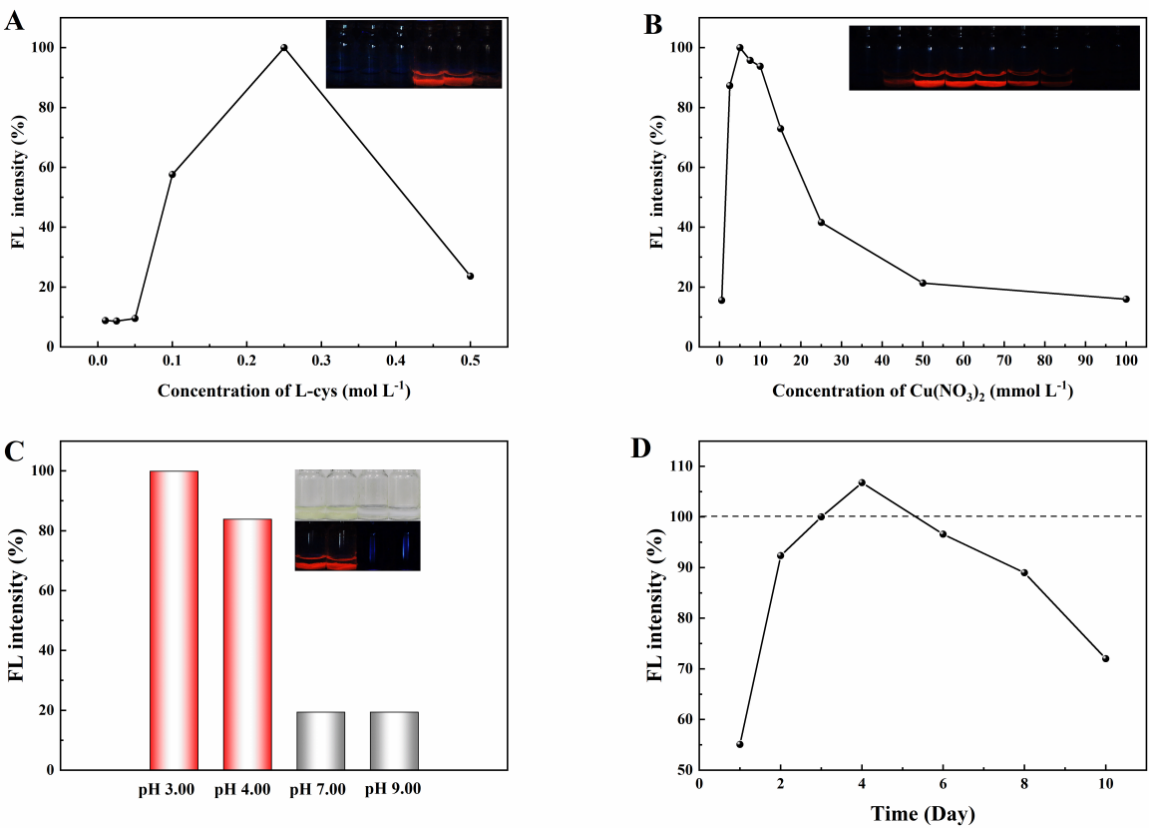
(**A**) Effect of the concentration of L-cysteine. (**B**) Effect of the concentration of Cu (NO_3_)_2_. (**C**) Effect of solution pH and (**D**) the stability of the fluorescence of the copper clusters. Photograph inserted: copper clusters at different conditions under 365 nm UV irradiation (The dashed line indicates the demarcation threshold at 100% fluorescence intensity).

To optimize the fluorescence properties of copper clusters, the synthesis conditions, including concentrations of L-cysteine, Cu (NO_3_)_2_, pH, and stability of fluorescence in copper clusters, were investigated. It can be observed from Figure 2A,B that the fluorescence intensity reaches the maximum with 0.25 mol L^−1^ L-cysteine and 5.0 mmol L^−1^ Cu^2+^. Considering that L-cysteine cannot be completely dissolved when its concentration exceeds 0.20 mol L^−1^, 0.10 mol L^−1^ L-cysteine and 5.0 mmol L^−1^ Cu^2+^ were selected for subsequent experiments. The copper clusters obtained by direct synthesis have a pH value of 3.00. Therefore, the influence of solution pH values (3.00, 4.00, 7.00, and 9.00) on the copper clusters was investigated (Figure 2C). As shown in the figure, the copper clusters exhibited red fluorescence at pH 3.00 and 4.00, whereas no fluorescence was observed at pH 7.00 and 9.00, which is consistent with previous reports [26]. The reason for this is likely fluorescence quenching caused by the hydrolysis of copper ions under high pH conditions. Consequently, subsequent experiments were conducted in the as-prepared aqueous solution without pH adjustment. In addition, the stability of the fluorescence of the copper clusters was also investigated in the experiment (Figure 2D). The results indicated that the copper clusters could remain stable for more than one week when stored at room temperature and protected from light.

### 2.3. Detection of Formaldehyde

**Figure 3:**
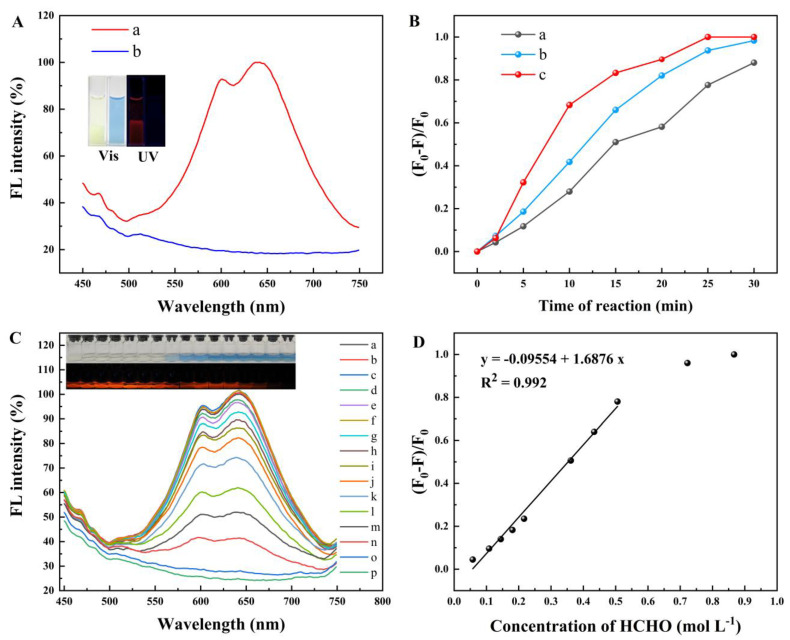
(**A**) Fluorescence spectra of copper clusters before (a) and after (b) react with formaldehyde (Insert: picture of copper clusters before and after react with formaldehyde under visualization and ultraviolet light). (**B**) Fluorescence spectra of optimization of detection conditions for formaldehyde (a, b, and c are concentration of HCHO: a = 72.22 mmol L^−1^, b = 180.62 mmol L^−1^, and c = 361.25 mmol L^−1^). (**C**) Fluorescence responses of different formaldehyde concentrations including a–p (Insert: corresponding to the pictures of the Cu clusters added with different concentrations of formaldehyde under visualization and ultraviolet light). (**D**) Standard curve of detecting formaldehyde.

Figure 3A showed that fluorescence was quenched when formaldehyde was added and no red color was discovered under UV light (the insert picture), indicating that the copper clusters can be applied for detecting formaldehyde. Figure 3B illustrates the impact of formaldehyde reaction time on the fluorescence of copper clusters, showing that the fluorescence signal of copper clusters was completely quenched within 30 min by different concentrations of formaldehyde. To reduce the reaction time and speed up the reaction process, a reaction time of 15 min was chosen. The sensitivity for detecting formaldehyde was evaluated and is shown in Figure 3C; the fluorescence intensity decreased with an increase in formaldehyde concentration from 7.22 mmol L^−1^ to 867.00 mmol L^−1^ (a-p: 0.0, 7.22, 14.45, 36.12, 57.80, 72.25, 108.38, 144.50, 180.62, 216.75, 289.00, 361.25, 433.50, 505.75, 722.50, 867.00 mmol L^−1^, respectively). A quantitative relationship could be established between the degree of fluorescence quenching of copper clusters and formaldehyde concentration; the fluorescence intensity showed a linear relationship with decreasing formaldehyde concentration ranging from 57.80 mmol L^−1^ to 505.75 mmol L^−1^ with a correlation coefficient of 0.992 (Figure 3D). The limit of detection (LOD, *n* = 11) for formaldehyde was calculated to be 17.06 mmol L^−1^.

**Figure 4:**
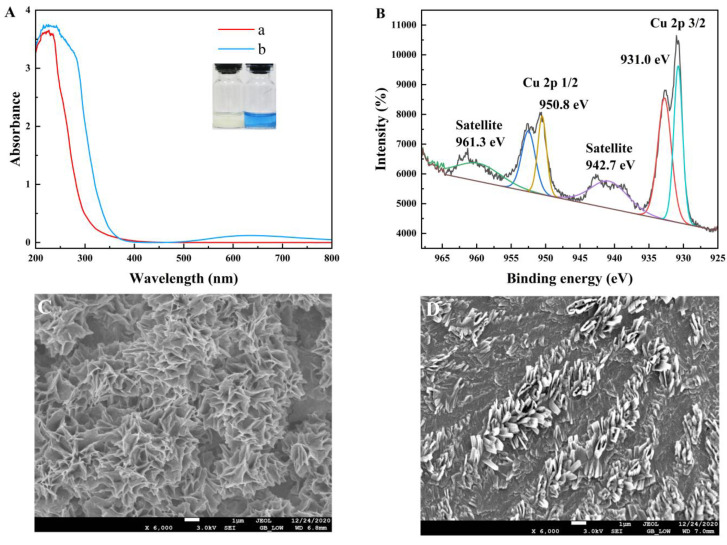
(**A**) UV-Vis spectra of copper clusters before (a) and after (b) react with formaldehyde. (**B**) High-resolution XPS spectrum of Cu 2p copper clusters after reacting with formaldehyde. SEM images of Cu clusters before (**C**) and after (**D**) reacting with formaldehyde.

The ultraviolet absorption spectrum of copper clusters after reacting with formaldehyde is shown in Figure 4A. A new ultraviolet absorption peak was observed around 640 nm, and the solution turned blue after the reaction. A comparison of the SEM images of copper clusters before and after the reaction with copper clusters (Figure 4C,D) revealed that the addition of formaldehyde disrupted the aggregated structure of the copper clusters. XPS characterization analysis was also conducted on the copper clusters after the reaction with formaldehyde, and the results for the Cu 2p characteristic peaks are presented in Figure 4B. The peaks at 924.9 eV and 961.5 eV correspond to the characteristic peaks of Cu(II), indicating that the addition of formaldehyde led to the oxidation of the exposed metallic cores Cu(0)/Cu(I) to Cu(II) by air, and this process was irreversible.

### 2.4. Selectivity

**Figure 5:**
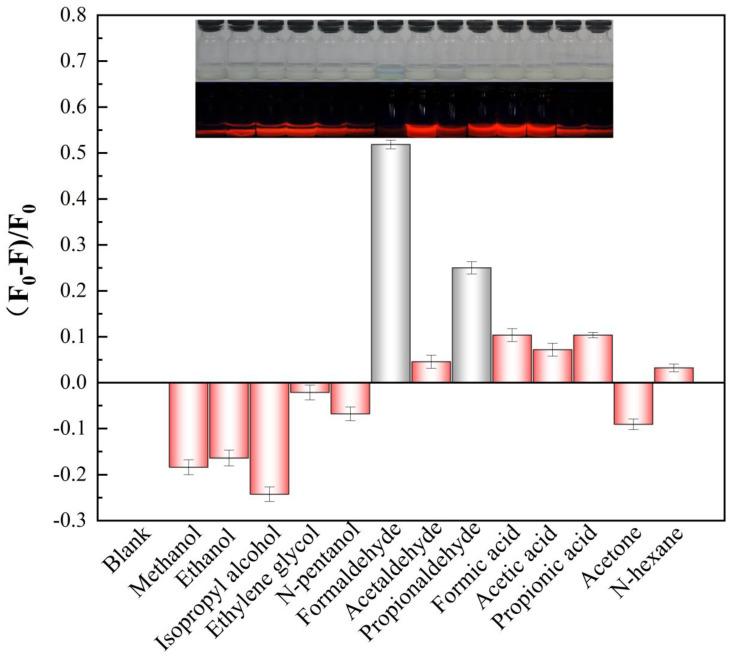
Selectivity of copper clusters to different VOCs. The photographs inserted are copper clusters interacting with formaldehyde interferents under visualization and under a 365 nm UV lamp.

The selectivity of this method was evaluated by testing the response to other VOCs in Figure 5. The fluorescence intensity changes of copper clusters were detected in the presence of interfering compounds including formic acid, acetic acid, propionic acid, acetaldehyde, propionaldehyde, acetone, N-hexane, methanol, ethanol, isopropyl alcohol, n-pentanol and ethylene glycol which were, respectively, added into copper clusters under the same conditions. The results demonstrate that common volatile organic compounds that may coexist with formaldehyde do not exhibit quenching effects on the fluorescence of copper clusters, except for propionaldehyde. This indicates that the method exhibits good selectivity. However, the quenching rate of propionaldehyde was not as high as that of formaldehyde, and the color of the solution did not change after the reaction of propionaldehyde with copper clusters. Therefore, they can be distinguished by limiting the reaction time or by using naked-eye observation as a second dimensional signal.

### 2.5. Detection of Formaldehyde in Air Samples

To evaluate the accuracy of this method, the presence of formaldehyde in indoor air samples was determined using the standard addition recovery method, with the results presented in Table 1. The recovery rates of formaldehyde samples ranged from 90% to 110%. Additionally, the fluorescence visualization results of the samples in Table 1 were consistent with the concentration ranges corresponding to the photographs in the standard curve. The results indicate that this method is applicable for the determination of formaldehyde in real samples.

### 2.6. Visual Detection of Formaldehyde

**Figure 6:**
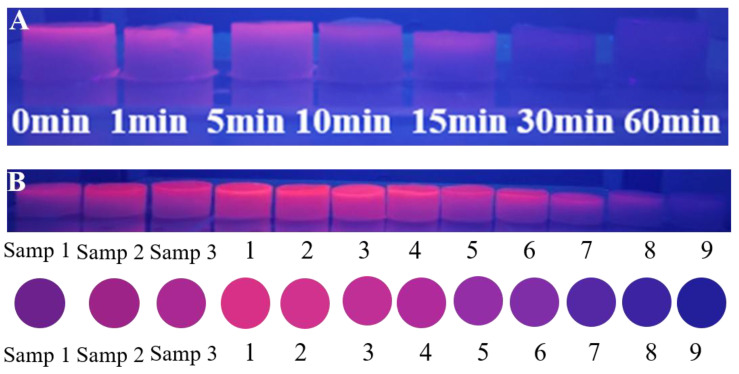
(**A**) Time optimization of the effect of 0.5 mol L^−1^ formaldehyde on hydrogel-stabilized copper clusters under 365 nm UV light. (**B**) Visualization of hydrogel-stabilized copper clusters for formaldehyde detection under 365 nm UV light.

The development of solid-state fluorescent materials not only enhances stability but also facilitates portable and visual detection. Therefore, in this work, hydrogel-stabilized L-cys-Cu clusters were prepared, and their effectiveness in the visual semi-quantitative detection of formaldehyde was investigated. Firstly, the hydrogel-stabilized L-cys-Cu clusters were placed in a 0.5 mol L^−1^ aqueous formaldehyde solution, and the fluorescence quenching of the formaldehyde was investigated at immersion times of 0, 1, 5, 10, 15, 30, and 60 min, respectively. As shown in Figure 6A, under irradiation by a 365 nm ultraviolet lamp, the red fluorescence of the hydrogel-stabilized L-cys-Cu clusters gradually faded with increasing immersion time, and complete quenching was achieved after 30 min of immersion. Therefore, a reaction time of 30 min was selected for subsequent experiments. Subsequently, when the fluorescent hydrogel-containing copper clusters was immersed in formaldehyde solutions of different concentrations, a gradual transition from intense red fluorescence to complete colorless state was observed, as illustrated in Figure 6B (concentration ranges of formaldehyde 1–9: 0.0, 0.0–0.02, 0.02–0.05, 0.05–0.10, 0.10–0.20, 0.20–0.30, 0.30–0.40, 0.40–0.50 mol L^−1^). This visual gradient enables semi-quantitative determination of formaldehyde concentration through direct color comparison, which is also consistent with the results obtained after RGB image processing. The accuracy of this visual detection method was validated using three simulated formaldehyde solutions with concentrations of 0.30 mol L^−1^ (sample 1), 0.15 mol L^−1^ (sample 2), and 0.075 mol L^−1^ (sample 3), respectively. As shown in Figure 6B, after adding the samples, the fluorescence color range of the hydrogel corresponded precisely to the concentration gradients of the samples, demonstrating that this method enables the convenient and intuitive semi-quantitative detection of formaldehyde.

## 3. Materials and Methods

### 3.1. Chemicals and Materials

L-cysteine, copper nitrate [Cu (NO_3_)_2_·3H_2_O], formaldehyde solution (40%), agarose, formic acid, acetic acid, propionic acid, acetaldehyde, propionaldehyde, acetone, n-hexane, methanol, ethanol, isopropanol, n-pentyl alcohol, and ethylene glycol were purchased from Kelong Chemical Reagent Factory, Chengdu, China. All reagents are of analytical grade or better. The formaldehyde samples were collected from the indoor air of the laboratory using a gas collection apparatus. Ultrapure water (18.2 MΩ·cm) was used with a purification water system (PCWJ-10, Pure Technology Co. Ltd., Chengdu, China).

### 3.2. Instrumentation

All fluorescence measurements were performed on a 970 CRT Fluorescence spectrophotometer (INESA Analysis Co., Ltd., Shanghai, China), the excitation and emission slits were set at 5 nm bandpass. UV-Vis absorption spectra were recorded by a UV-1200 spectrophotometer (Mepuda Instrument Co., Ltd., Shanghai, China). The morphology of the materials was analyzed by using a JEOL-JSM-7500F Field Emission Scanning Electron Microscopy (Nippon Electronics Co., Ltd., Aki City, Tokyo, Japan). Elemental and functional groups analysis were obtained by a IR200 Fourier transform infrared (FT-IR) spectrometer (Thermo Fisher Scientific, Waltham, MA, USA) and the X-ray photoelectron spectroscopy (XPS) measurements were preformed using an ESCALAB 250Xi instrument (Thermo Fisher Scientific, Waltham, MA, USA) with Al Kα X-ray radiation (1486.6 eV) as the excitation source, and C1s (284.8 eV) was used as the reference energy for all binding energies. Indoor formaldehyde gas was collected by an indoor air quality detector with ultrapure water as the absorption solution (Sild Indoor Environmental Monitoring and Management Co., Ltd., Wuxi, China).

### 3.3. Synthesis of Copper Clusters

The synthesis of Cu clusters was improved based on previous work [26]. In a typical experiment, the copper clusters were prepared by adding an aqueous solution (1 mL) 0.2 mol L^−1^ L-cys into a 0.01 mol L^−1^ Cu (NO_3_)_2_ aqueous solution (1 mL) at room temperature. Following shaking to make it full reaction, it was then stored at room temperature in the dark for 24 h to form fluorescent copper cluster aggregates.

### 3.4. Procedures for Fluorescence Measurements of Formaldehyde

A series of different volumes of formaldehyde solutions (40%) were pipetted with a microinjection needle and added to the Cu cluster solution. The mixture was used for fluorescence measurements after 15 min of reaction. To evaluate the selectivity of the proposed method, 50 μL of formic acid (0.569 mol L^−1^), acetic acid (0.424 mol L^−1^), propionic acid (0.324 mol L^−1^), acetaldehyde (0.175 mol L^−1^), propionaldehyde (0.331 mol L^−1^), acetone (0.330 mol L^−1^), n-hexane (0.181 mol L^−1^), methanol (0.599 mol L^−1^), ethanol (0.417 mol L ^−1^), isopropanol (0.318 mol L^−1^), n-pentyl alcohol (0.213 mol L^−1^), and ethylene glycol (0.435 mol L^−1^) were investigated. The solutions were mixed thoroughly and left for 15 min, followed by fluorescence spectroscopy at an excitation wavelength of 400 nm.

### 3.5. Preparation Process for Real Samples

Indoor air samples were collected using a gas collection device. The samples were dissolved in 4 mL of ultrapure water at a flow rate of 500 mL/min for 20 min. Then, 50 μL of the absorbent solution was pipetted and added to the Cu clusters for fluorescent measurements. Determined concentrations of formaldehyde solutions (72.25, 144.50 and 289.00 mmol L^−1^) were used for the spiking method.

### 3.6. Preparation of Hydrogel-Stabilized Copper Clusters

Weigh 0.2000 g of agarose and dissolve the agarose in 10 mL of ultrapure water, heat the solution until boiling, and then allow the agarose solution to cool until it reaches 50 °C. Transfer 1 mL of the previously prepared copper cluster solution into the agarose solution, stir rapidly and uniformly, and then solidify within the mold to form the desired shape.

## 4. Conclusions

To conclude, we established a fluorescent assay based on L-cys-Cu clusters for formaldehyde measurement. The preparation of copper clusters without additional reagents is environmentally friendly and leads to easy destruction in the presence of formaldehyde, which resulted in the quenching of fluorescence and generated obvious color changes for visual readout. This method is simple to operate, selective, and is expected to be applied for the rapid detection of formaldehyde in indoor air monitoring.

## Figures and Tables

**Table 1:** Analytical results for formaldehyde in simulated samples using the proposed method.

Sample	Added (mmol L^−1^)	Detected ^a^ (mmol L^−1^)	Visualize
1	0	nd	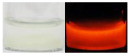
2	72.25	70.08 ± 0.01	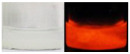
3	144.50	133.90 ± 0.01	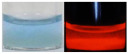
4	289.00	301.12 ± 0.02	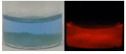

^a^ = Average standard deviation (*n* = 3); nd = not detected.

## Data Availability

The original contributions presented in this study are included in the article. Further inquiries can be directed to the corresponding author.
